# The History of Mind Cure

**Published:** 1888-08

**Authors:** Helen Densmore


### THE HISTORY OF MIND CURE: AN ANALYSIS FROM A PHYSIOLOGICAL STANDPOINT.

BY DR. HELEN DENSMORE.

### PART I.

When I began to practice the art of healing I was appalled at the failure to find anything approaching certainty in the practical application of the science as taught in the schools ; and I, as scores of others have done, should have forsaken it but for the application of a principle laid down in the best text-book on pathology, to wit : "That all pathological changes (which means diseased conditions) are caused by impaired nutrition." I saw that if this be true, it offers the philosopher's stone in matters of health ; the "open sesame" to the enchanted isle, a sure and safe harbor for ninety-five per cent. of the pilgrims in quest of the desired Mecca,—restored health.

Investigation into the subject of nutrition and its relation to health and disease, convinced Dr. Densmore and myself that the diet of civilization bears the same relation to the manifold forms of disease in modern life that stimulating drinks bear to delirium tremens ; and so surely as a mania-á-potu cannot exist without such drinks, the various forms of chronic disease that are devouring the race would disappear under the proper regimen—and that it would be as impossible for mankind to be ill if they lived hygienic lives as for men and women to have delirium tremens without stimulating drinks.

With this key we proceeded to prove the truth of this principle. We found ample confirmation in its application, in a large private practice. Having, as we believed, the certainty of science, we fearlessly announced our platform,—that health is man's birthright ; that it is as natural to be well as to be born ; and wherever it was tested by an obedience to our direction as to hygienic habits, and especially to diet, lo ! we found as a result sure to follow—restored, or greatly improved, health. We never met with failure where the curative action, often brought about by the system when the change of diet is made, was understood and patience with the action practiced. In our own persons we came to know the certainty of health at all times, under all circumstances ; and with others in proportion as they approximated to the prescribed rules of life.

We had been practicing this method, and teaching it to all who would learn, for years when I first heard of the claims of Mind Cure healing.

It came to me through a bright lady patient in Washington, D. C., and the account which she gave me of it, information acquired from one of its ablest exponents, Mrs. Stuart, of Boston, at once claimed my attention and challenged my investigation. I had long regarded illness as the hydra-headed monster of modern social life, and the profession of healing the sick being with me well-nigh a religion, any new thought that offered help was welcomed.

The first introduction to the philosophy underlying this new and strange dispensation of health was indeed quite sufficient to hurry me on. I was told that there is no such thing as sickness, pain or death; that these conditions are the result of *erroneous judgment, false mental modes*, and exist in the mind alone. When I asked, is there no physiological law to which we are answerable for violating its conditions? I was told there is no physiology, no organic processes, no functional activity. These are all modes of *mental* activity; *all is in the mind*; matter has no existence save as an *idea in mind;* muscular force is mental force; all functional action, prehension, mastication, deglutition, digestion, assimilation and excretion are performed by thought; in short, there is no physiology; and pain and suffering, sin and death, are in the world because of a belief in the mind that they exist. A proper understanding of this truth dispels the mistake, and sin and suffering disappear. Then, I said, it is a mistake that we have liver, lungs, heart, etc. Yes, was the reply, they are only reflections of thought. They have no existence in reality; they are, when diseased, only photographs of the distorted thoughts which have taken the place of perfect ones, which would in place of this distortion give us a picture of health, and this without reference to material conditions of any kind.

I could have listened to all this as metaphysical abstractions, remembering Berkely and his school of philosophy, and allowed it to pass as belonging to the incomprehensible realm, but for the fact that a decidedly practical side to this picture appeared in the marvels of cure that were related and apparently easy of corroboration, and which, upon investigation, I verified.

I had been a believer in many "modern miracles" in healing, claimed to be done by *supersensual power*, forced to believe by occular demonstration and also by unimpeachable human testimony, for some time; but there is a clear road out of this dilemma to one who really believes in the supremacy of spirit and the power of divine influx, for all these methods claim for ther source divine power in answer to prayer. I chanced to read some ten or a dozen years ago "Our Lady of Lourdes," a Roman

Catholic legend giving details of many miraculous cures performed in a far-off French province by "Our Lady of Lourdes," a canonized saint of the church of our own day. Several years later the Rev. Dr. Tyng preached a sermon in St. Thomas Church in New York City on "Modern Miracles," giving his personal experiences in visiting this remarkable grotto presided over by this saint, and where many invalids had sought and found healing from supposed incurable physical conditions of illness and deformity. Dr. Tyng was himself cured of some malady which medical science had failed to relieve.

So, as I say, I had given my adherence to the possibility of supersensual healing, and was not surprised that such things happen, but I was bewildered that these cures were said to be made by a system of mental healing that could be taught; that it claimed to be a science; that once grasped, one could demonstrate the truth of this science to one's self, and also others; that it was considered in no sense a gift, but an understanding of a truth, which to comprehend is all that is required. I was told also that it bears no relation to the faith cures; that it requires neither faith on the part of the patient, nor peculiar conditions of sympathy or *rapport* between the patient and healer; that it ignores physiological law, diet, exercise and hygiene as remedial agents, which if proven to be true, entirely destroys our new gospel of health, to be established upon natural law, and that, too, by a system so much more easily operative upon the hearts and minds of those who have acquired the artificial habits of civilization.

It was this scientific claim that staggered me, and seeking Mrs. Stuart of Boston, I asked the privilege of making some enquiries, to which she kindly consented.

Do you claim this system of Mental healing as a science?

Can it be taught?

Do you answer all questions upon the apparent occult nature of this truth scientifically, and not by asserting its spiritual nature, and hence not explainable on scientific principles? To all these questions Mrs. Stuart answered most emphatically, yes.

I entered my name for a course of Mrs. Stuart's instruction, for which a class was about to be formed, but, for some reason, I was not informed when the class was completed, and so was disappointed in not being able to take Mrs. Stuart's lessons personally.

Not discouraged by this failure, (and it must be remembered that four years ago it was not as easy to enter upon the study of Mind Cure as it is to-day, when teachers and schools are everywhere to be found), I persevered until I found a chance to study it. In addition to taking two courses of lessons of a very able student of Mrs. Newman, who gave the same teaching as Mrs. Newman herself, I obtained access to very complete notes of the twelve lessons of Mrs. Stuart, as given to her pupils ; I have read and studied the works of Dr. Evans, a prolific, noteworthy and able author in this field. I took a course of lessons from Mrs. Sarah Stanley Grimké, I had two long interviews with Mrs. Eddy, whose book, "Science and Health," I have read and carefully studied. My investigations and study cover about three years, largely absorbing my mind during that period.

In regard to the different schools of mind cure, so far as I am able to see, in essential principle and method of demonstration, they are all offshoots and repetitions of Mrs. Eddy's teaching, notwithstanding the fact that misunderstandings and dissensions have divided them into various and ofttimes *hostile* camps.

The teachers whose courses of instruction I have given are from the early harvests of this new philosophy, now called by its founder "Christian Science." Mrs. Stuart and Mrs. Newman were among the first who branched off from the original plant, and being women of unusual force of character and mental power, they have impressed their personality most forcibly upon the public mind.

At first Mrs. Eddy was looked upon as the sole authority and repository of this truth, and she claims almost papal infallibility in her personal teachings. Gradually this hold was loosened, through what influence it is needless to consider, and these two teachers were in turn looked upon by their respective students as representing the law and the prophets. But the sphere of this newly discovered truth began to widen as pupils increased in numbers.

At first pupils were required to obligate themselves not to teach until they had spent two years in practically demonstrating this system of healing. Gradually these restrictions were disregarded, and the number of teachers and healers greatly increased and the fees considerably decreased ; so that now this new school of mental healing has representatives in nearly every city, town and village throughout the country.

One of the most remarkable women developed by this movement is Mrs. Sarah Stanley Grimké. Mrs. Grimké was healed by Mrs. Stuart, and almost immediately began a most wonderful unfoldment of her own powers as a teacher of metaphysical and spiritual lore. This lady formulated the teaching into a statement, which her teacher at once pronounced worthy to be used as a text-book by teachers. The book, called "Personified Unthinkables," an argument against physical causation, attracted attention outside the ranks of modern metaphysicians as a noticeable work in mental science. Her second work, "First Lessons in Reality," led off into a mystical realm dealing largely in symbolism, and consequently has not succeeded in gaining that confidence of the Mind Cure public which it ought to command. However, Mrs. Grimké must be counted as among the most profoundly phenomenal outgrowths of the movement. She is a young woman of charming personal appearance and remarkable powers as an exponent of spiritual truth.

Mr. Colville, of Boston, has perhaps reached a larger number of persons than any one teacher of Mind Cure. He has not put a limit to the number of pupils, has charged small fees and made his lessons less of a specialty than other teachers, confining them more particularly to the philosophy of spirit domination in all directions. Mr. Colville is an inspirational speaker and hails from the ranks of modern Spiritualism, differing in this particular from all others; as opposition to this belief in all its forms and phases is one of the tenets of the Mind Cure cult.

Dr. J. W. Dewey, of Buffalo, as a teacher and writer in this new faith has attracted attention. "The Theosophy of the Christ," an introduction to a larger work just published, called "Christian Theosophy," is one of the best works upon this subject. Unlike the original school of Christian science, Dr. Dewey recognizes the body; does not ignore physiology; and, consequently, is not received into free communion with the true saints.

J. W. Swartz, of Chicago, is at the head of a school, started early in the history of this movement, and has done valiant work for the cause. He started the first magazine out of Boston, and the first propoganda that reached out to the public; he has evinced a spirit of fraternal love and interest in all schools and individuals; thus embodying, in his life and practice, the teaching and example of Christ.

I cannot pass from this part of my subject, without mentioning C. M. Barrows, of Boston as a writer upon this subject. "Bread Pills:" What It is, and How it is Done," by this accomplished author was the first popular practical statement of Mind Cure that appeared upon the foggy atmosphere of this vague subject. This book was published in 1886 and proved a vertible ray of light to many gropers in the dark for the whys and wherefores of Mental Science, as applied to the healing of the sick. Later he has published "Facts and Fallacies of Mind Cure." This book is full of fervor for the truth of the principle, of lofty aspirations after its highest manifestation, and valuable criticism for some of the follies of its teaching.

It would be strange indeed if, in the rise and spread of so new and strange an innovation, there should not be fungus, mushroom growths upon the plant. The intense commercial spirit of the age generates an artificial heat that seeks through all possible channels for that upon which it can act. It forces growth abnormally in every direction of gain, and its influence is felt in this movement as in every one that touches the civilization of the age. So we see "Colleges" and "Institutions" organized by clever advertising methods that would do credit to the most exaggerated claims to patent nostrums, mechanical inventions, and stock jobbing schemes. "This is the way, the only way," these advertisers tell us; "if you come up by any other, you will fail to reach the desired goal. If you have failed with others, come to us! We are the only ones who have the unadulterated science without fallacy or flaw."

But in spite of all the drawbacks, all the crudities and inconsistencies of the ignorant and overzealous workers as well as the fundamental weakness of its claims, as formulated and taught by its ablest exponents, the growth of this system, during the past year, has been phenomenal. In September there was held a convention in Boston, the attendance at which was large, and the interest manifested the intellectual quality of those who made up the audiences, as well as a prophecy of still larger future increase. And while there are dissensions among the different schools, they are all based on the foundation stone of the supremacy of spirit; and at this meeting all came forward, for the time forgetting their differences, and joined in communion and conference on this great theme.

There is one feature of this wave, or revival, if it may be so styled, of the Christian methods of old, which has exacted comment and often criticism; it is the large price that has been charged for the lessons. From the first it has seemed to be a most gracious dispensation to the wealthy classes. In the past, new dispensations of spiritual truth have come first to the lowly. The despised Nazarene, the master whom Christian Science claims, had nowhere to lay his head; and the gospel he offered was without money, and without price. The modern mystics have erected their altars within the charmed circles of luxury; brown stone fronts have opened their doors for the high priestess of Christian Science to declare the glory of the real man, and the nothingness of material things. So, too, the fashionable world has not turned an altogether deaf ear to its pleadings; and clergymen of the various established orders have often assumed only a half patronizing protest against the innovation.

Thus it has seemed to come as a special blessing and in a guise that many from the conventional and conservative world have been able to receive it, and the high price that has been charged for it has made this condition, or been largely the cause of this attention on the part of the upper layers of the social structure ; and as a result we see in this quarter quite a new thought born in regard to the nature of illness and the attitude to be presented toward it. To be taught that there is no illness, that its appearance is to be denied, ignored, never referred to ; that all evil conditions are but appearances, without reality and controlled by thought, has had and must have beneficial effect upon sin and sickness, and quite changes the view of death. In so far as this thought has found lodgement in the heart, and expression in the life, through the teaching of Mind Cure, it has been a benefaction and a benediction.

But there is altogether another side to all this matter, and, as I said at the outset, I propose to show in this exposition wherein, in my judgment, this system, *as formulated and now taught,* although containing a great and important truth, is destined to do more injury in encouraging license in the matter of diet, in intemperate living, in ignoring the physiological nature of man, and in teaching contempt for hygiene and an orderly physiological life, than all the good accomplished by its phenomenal cures. (*To be continued.*)

